# Impacts of mild and severe COVID-19 on sick leave

**DOI:** 10.1093/ije/dyab182

**Published:** 2021-08-30

**Authors:** Katrine Skyrud, Kjetil Telle, Karin Magnusson

**Affiliations:** 1 Norwegian Institute of Public Health, Cluster for Health Services Research, Skøyen, Oslo, Norway; 2 Faculty of Medicine, Department of Clinical Sciences Lund, Orthopaedics, Clinical Epidemiology Unit, Lund University, Lund, Sweden

Using an observational pre-post design with a comparison group based on individual-level data from the Norwegian Emergency Preparedness Register,^1^ we aimed to study sick leave among all Norwegian residents aged 20 to 70 years with an employment contract, who were tested for SARS-CoV-2 from 1 March 2020 to 1 February 2021 [*N* = 1 177 274 with mean (SD) age 40 (13) years, 46% men).

We constructed our data as a panel and used a difference-in-differences design^2^^,^^3^ to contrast doctor-certified sick leave (any cause) before and after testing, across employees with negative test and (i) positive test without being hospitalized (mild disease) and (ii) positive test with hospitalization (severe disease) by groups of age and sex.^4^^,^^5^ We estimated the percent relative difference in change in health care use from 3 months before to 6 months after testing, i.e. comparing the difference in change over time for persons with mild disease (*N* = 33 761) vs persons with no disease, as well as for persons with severe disease (*N* = 1168) vs persons with no disease.

Men and women aged 20–44 and 45–70, with mild and severe COVID-19, had substantially elevated sick leave at 1–4 weeks following a positive test, and the elevation gradually disappeared at 5–8 weeks, 9–12 weeks, and 16–24 weeks (Figure 1, Table 1). From 5–8 weeks after test and onwards, the elevated sick leave following COVID-19 differed by age, sex and severity of initial disease. Women aged 45–70 years with mild disease had more prolonged elevation in sick leave than their younger counterparts (Table 1). Women’s impact on sick leave was not more prolonged after severe disease than after mild disease (Table 1). In contrast for men, there were fewer age differences and disease severity (i.e. severe disease) seemed to be their main determinant of elevated sick leave rates following COVID-19 (Table 1).

**Figure 1:**
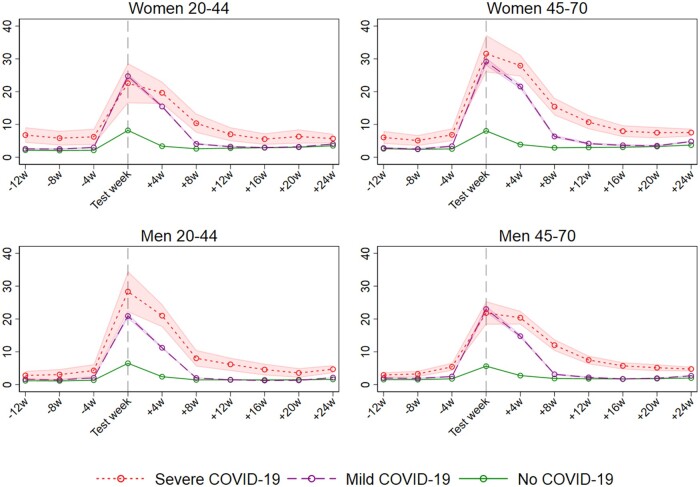
Estimated rates of weekly [95% confidence interval (CI)] received doctor-certified sick leave from 12 weeks before to 24 weeks after week of polymerase chain reaction test for SARS-CoV-2, for severe COVID-19, mild COVID-19 and no COVID-19 for different sex and age groups. Estimates adjusted for comorbidities, birth country and calendar month

**Table 1:** Difference-in-differences (DiD) estimates of impacts of COVID-19 on doctor-certified sick leave from 12–1 weeks before polymerase chain reaction test for SARS-CoV-2 to 1–4, 5–8 and 9–12 weeks after as well as 13–24 weeks after polymerase chain reaction tests

	Women 20–44		Women 45–70		Men 20–44		Men 45–70	
Weeks before PCR test	B (95% CI)	% relative diff. (95% CI)	B (95% CI)	% relative diff. (95% CI)	B (95% CI)	% relative diff. (95% CI)	B (95% CI)	% relative diff. (95% CI)
Mild disease								
1–4	11.5 (11.1, 11.97)	508 (488, 528)	17.3 (16.6, 18.0)	646 (620, 673)	8.33 (8.0, 8.67)	684 (657, 711)	11.49 (11.0, 12.0)	708 (676, 740)
5–8	0.9 (0.6, 1.19)	40 (27, 52)	3.1 (2.6, 3.5)	114 (97, 131)	0.07 (−0.1, 0.25)	5 (−10, 21)	0.76 (0.5, 1.1)	47 (29, 65)
9–12	−0.1 (−0.4, 0.13)	−6 (−17, 6)	0.8 (0.4, 1.2)	29 (15, 43)	−0.49 (−0.7, −0.32)	−40 (−54, −26)	−0.08 (−0.3, 0.2)	−5 (−21, 11)
16–24	−0.2 (−0.4, 0.04)	−7 (−16, 2)	0.5 (0.3, 0.8)	20 (9, 30)	−0.15 (−0.3, −0.01)	−12 (−23, −1)	0.02 (−0.2, 0.2)	1 (−11, 14)
Severe disease								
1–4	12.2 (8.5, 15.8)	537 (375, 698)	20.6 (17.1, 24.2)	771 (638, 904)	16.5 (12.9, 20.1)	1353 (1059, 1647)	15.4 (13.3, 17.5)	948 (819, 1078)
5–8	3.7 (0.5, 6.9)	162 (21, 303)	9.1 (6.2, 12.0)	340 (232, 448)	4.4 (1.9, 7.0)	365 (152, 577)	7.9 (6.1, 9.7)	488 (379, 597)
9–12	0.1 (−2.4, 2.7)	6 (−107, 119)	4.3 (2.0, 6.6)	160 (73, 247)	2.6 (0.4, 4.7)	211 (32, 389)	3.5 (2.0, 4.9)	213 (126, 300)
16–24	−1.7 (−3.8, 0.3)	−77 (−168, 15)	0.5 (−1.0, 2.1)	20 (−38, 79)	0.8 (−0.7, 2.4)	69 (−57, 194)	0.7 (−0.2, 1.6)	42 (−14, 98)

The DiD estimates (B) capture the change in health care use from 12–1 weeks before polymerase chain reaction test to 1–4, 5–8 and 9–12 weeks after as well as 13–24 weeks after polymerase chain reaction test for patients with mild or severe COVID-19 compared with the change over the same period for patients for patients with no COVID-19, by age and sex groups

To our knowledge, this study is the first to explore short- and long-term impacts of COVID-19 on sick leave for employees. Important strengths of our study are the prospective design and use of data that include all employees who were tested for SARS-CoV-2 in an entire country. Our findings are representative for countries with equal access to health care and universal and generous unemployment and sick leave insurances. When interpreting these findings, it is important to bear in mind that the pandemic develops over time, i.e. the effect of contracting the virus is likely different for different time periods and regions depending on factors such as immunity in the population, testing criteria and access, vaccination etc. As an example, as more people get vaccinated, as fewer get severe disease and as health care systems have more knowledge of how to handle the disease, it might be assumed that the impact of COVID-19 on sick leave will decrease in the future. We propose this topic, as well as the extent to which the impact on sick leave is heterogeneous for different occupation and industry codes, as a topic for future study.

Several important limitations are worth mentioning. First, layoffs during the pandemic may have led to a change in the composition of the workforce. To explore this more in detail, we have assessed the potential presence of a healthy worker selection bias by studying any primary care use among everyone during their working age, with similar findings.^5^ Second, we had no information regarding self-certified sick leave. However, we expect the impact of only including doctor-certified sick leave to be minimal given the required 10–14 days’ quarantine following a positive test for SARS-CoV-2. Third, the subgroup estimates of sick leave rates following severe COVID-19 (Figure 1) indicate that the common pre-trend assumption of differences-in-differences estimates may not fully hold in some subgroups. One should thus, and as always in observational studies, be careful in making too strong causal interpretations of, especially, the long-term effect estimates of mild and severe disease on sick leave.

In conclusion, the impact of COVID-19 on sick leave depended on age for women (2 vs 3–6 months elevation for women aged 20–44 vs 45–70 years, respectively). For men, the impact depended on disease severity (1–2 vs 3 months elevation for men with mild vs severe disease, respectively).

## Ethics approval

The establishment of an emergency preparedness register forms part of the legally mandated responsibilities of the Norwegian Institute of Public Health (NIPH) during epidemics. Institutional board review was conducted, and the Ethics Committee of South-East Norway confirmed (4 June 2020, #153204) that external ethical board review was not required.

## Funding

The study was funded by the Norwegian Institute of Public Health. No external funding was received.

## Data availability

Individual-level data of patients included in this manuscript after de-identification are considered sensitive and will not be shared.
